# BMI-stratified nomograms to predict early SIRS-defined sepsis after flexible ureteroscopy

**DOI:** 10.3389/fmed.2026.1855137

**Published:** 2026-07-16

**Authors:** Qing-Hua Luo, Da-Qing Zhu, Jun-Tao Tan, Lei-Hua Cao

**Affiliations:** 1 Department of Urology, Nanchang People’s Hospital, Nanchang, Jiangxi, China; 2 Department of Infectious Disease, Fifth People’s Hospital of Ganzhou, Ganzhou, Jiangxi, China; 3 Jiangxi Province Key Laboratory of Breast Diseases, Nanchang People’s Hospital, Nanchang, Jiangxi, China

**Keywords:** body mass index, flexible ureteroscopy, nomogram, risk prediction, SIRS-defined sepsis

## Abstract

**Background:**

Postoperative sepsis is a serious complication of flexible ureteroscopy (FURS). Body mass index (BMI) may modify risk, but BMI-stratified prediction models are lacking. We developed and internally validated BMI-specific nomograms for early SIRS-defined sepsis after FURS.

**Methods:**

This single-center retrospective cohort included 1,254 adults undergoing FURS (2020–2024), stratified by BMI (<25 vs. ≥ 25 kg/m^2^). Multivariable logistic regression identified independent predictors of SIRS-defined sepsis within 48 h in each stratum. BMI-specific nomograms were constructed and internally validated using 1,000 bootstrap resamples.

**Results:**

SIRS-defined sepsis occurred in 78/1,254 (6.2%): 42/504 (8.3%) with BMI ≥ 25 vs. 36/750 (4.8%) with BMI < 25 (*p* = 0.011). In both strata, positive urine culture, staghorn stones, CRP ≥ 10 mg/L, and procalcitonin ≥0.5 ng/mL were independent risk factors. Operative time ≥60 min was significant only in the BMI ≥ 25 group, while postoperative albumin ≥35 g/L was protective only in the BMI < 25 group. The BMI ≥ 25 nomogram showed excellent discrimination (AUC 0.94, optimism-corrected 0.92), and the BMI < 25 nomogram performed robustly (AUC 0.90, optimism-corrected 0.88), with good calibration and net benefit.

**Conclusion:**

BMI-stratified nomograms identified shared and BMI-specific risk factors for early SIRS-defined sepsis after FURS. These tools may aid individualized perioperative risk assessment, pending external validation.

## Introduction

Flexible ureteroscopy (FURS) is a common minimally invasive technique for upper urinary tract stones, offering reduced trauma, faster recovery, and high stone-free rates (1). However, postoperative sepsis remains a serious complication, with early systemic inflammatory response syndrome (SIRS)-defined sepsis occurring in 4–8% of cases, potentially leading to organ dysfunction, prolonged hospitalization, and increased mortality (2). Effective preoperative risk stratification tools are urgently needed.

Body mass index (BMI) is a well-recognized determinant of perioperative infectious complications (3). Obesity is linked to chronic inflammation and immune dysregulation, which may increase susceptibility to sepsis (4). During FURS, obesity can also complicate surgical access and prolong operative time, further elevating infectious risk (5). Conversely, lower BMI may indicate reduced physiological reserve due to malnutrition or sarcopenia, potentially impairing host defense mechanisms (6, 7). These observations support a BMI-stratified approach to risk assessment.

Despite the clinical relevance of BMI, few studies have systematically explored its modifying role in sepsis after FURS. Existing prediction models typically treat BMI as a single covariate, potentially obscuring subgroup-specific risk patterns. Moreover, many models do not incorporate widely available biomarkers such as C-reactive protein (CRP), procalcitonin (PCT), or postoperative albumin, and rarely report robust internal validation or decision-curve analysis, limiting their clinical utility (8, 9). Furthermore, the distinction between SIRS-based endpoints and Sepsis-3 criteria has not been consistently addressed in existing FURS prediction models, making it difficult to compare findings across studies (10).

Nomograms offer an intuitive and practical means of individualized risk prediction by integrating multiple clinical variables into a visual scoring system (9). A BMI-stratified nomogram approach may improve predictive accuracy and facilitate personalized perioperative management.

In this study, we developed and internally validated BMI-stratified nomograms to predict early postoperative SIRS-defined sepsis after FURS using a retrospective cohort of 1,254 patients. Our goal was to identify BMI-specific risk factors and to provide clinically actionable tools for risk stratification that can be applied at the point of care. Throughout this manuscript, ‘SIRS-defined sepsis’ refers to the presence of ≥2 SIRS criteria within 48 h postoperatively; this endpoint is distinct from Sepsis-3 criteria, which require evidence of organ dysfunction (11).

## Materials and methods

### Study design

This retrospective cohort study developed and internally validated BMI-stratified risk prediction models for SIRS-defined sepsis following FURS. Patients treated at Nanchang People’s Hospital between January 2020 and December 2024 were included. According to World Health Organization criteria (12), patients were stratified into BMI ≥ 25 kg/m^2^ and BMI < 25 kg/m^2^ groups.

### Data selection and definition of the SIRS-defined Sepsis endpoint

Inclusion criteria were: (1) age ≥18 years with upper urinary tract stones confirmed by computed tomography or kidney–ureter-bladder X-ray; (2) FURS as the primary procedure; (3) complete clinical data. Exclusion criteria: (1) bilateral or staged procedures performed in the same operative session; (2) lower urinary tract stones; (3) preoperative SIRS-defined sepsis or severe infection; (4) end-stage renal disease or immunosuppressive therapy.

Demographic, clinical, and perioperative variables were extracted from electronic records: age, sex, BMI (≥25 vs. < 25 kg/m^2^), smoking; comorbidities (diabetes, hypertension, hyperlipidemia, history of urinary tract infection [UTI]); surgical factors (operative time [<60 vs. ≥ 60 min], preoperative indwelling stent, stone characteristics [size, staghorn, location, number]); preoperative laboratory variables (urine culture [positive/negative], CRP [<10 vs. ≥ 10 mg/L], PCT [<0.5 vs. ≥ 0.5 ng/mL]). Postoperative serum albumin within 24 h (<35 vs. ≥ 35 g/L) was also recorded.

The primary endpoint was SIRS-defined sepsis within 48 h after FURS, defined as ≥2 of the following: (1) temperature <36 °C or >38 °C; (2) heart rate >90 beats/min; (3) respiratory rate >20 breaths/min or PaCO₂ < 32 mmHg; (4) white blood cell count <4,000/mm^3^ or >12,000/mm^3^, or >10% immature neutrophils (8, 13). SOFA/qSOFA components were not consistently available; therefore, Sepsis-3 criteria could not be applied (11). Throughout this manuscript, ‘SIRS-defined sepsis’ specifically denotes the composite endpoint described above and is not equivalent to, nor intended to imply, the Sepsis-3 definition of sepsis. The term ‘sepsis’ is used only when referencing the Sepsis-3 definition in the literature or when discussing the broader clinical complication in the context of prior studies.

### Perioperative antimicrobial management

Midstream urine culture was obtained preoperatively (typically within 7 days). Intravenous antibiotic prophylaxis was given within 60 min before incision, guided by institutional antibiogram and allergy history; re-dosing followed policy for prolonged procedures (14). For positive preoperative cultures, elective FURS was deferred for culture-directed therapy until clinical improvement (and, when feasible, negative repeat culture). In urgent infection with obstruction, initial drainage (ureteral stent or nephrostomy) plus antibiotics was performed, with FURS scheduled electively. For patients already on culture-directed therapy at surgery, the regimen was continued as prophylaxis (15). Because antibiotic choices varied retrospectively, only preoperative urine culture status (positive vs. negative) was included as a predictor.

Preoperative CRP and PCT were routinely obtained. If CRP ≥ 10 mg/L and/or PCT ≥ 0.5 ng/mL co-occurred with clinical signs of infection or positive urine culture, elective FURS was deferred. Isolated elevations without infection signs prompted repeat testing and individualized judgment; if surgery proceeded, standard prophylaxis with enhanced monitoring was used. PCT was included for its higher specificity for bacterial infection (16, 17).

### Statistical analysis

Analyses used R (v4.3.0). Patients were stratified by BMI (≥25 vs. < 25 kg/m^2^). Continuous variables were compared using t-test or Mann–Whitney U test; categorical variables using χ^2^ or Fisher’s exact test. For each stratum, multivariable logistic regression was fitted. Clinically essential predictors were pre-specified and retained regardless of univariate *p*-values (preoperative urine culture, staghorn stone, CRP, PCT, operative time; plus postoperative albumin for the BMI < 25 model). Univariable analyses were performed for all candidate variables, and the results are summarized in the Results section. Model size was constrained to achieve events-per-variable ≥7–8. Additional covariates with univariate *p* < 0.05 were considered while respecting EPV; multicollinearity was checked (all VIF < 2). Due to the retrospective nature of the data, missing values were minimal across all variables, and complete-case analysis was therefore performed. Models used complete cases for included variables. Model performance was assessed by AUC (with 95% CI via bootstrap) and calibration (calibration curve, Hosmer-Lemeshow, calibration slope/intercept). Bootstrap internal validation (1,000 resamples) provided optimism-corrected AUC and calibration metrics, plus bias-corrected calibration plots. Decision-curve analysis (DCA) quantified net benefit across threshold probabilities 0.10–0.70. Discrimination, calibration, and DCA were prespecified primary metrics. Two-sided *p* < 0.05 was significant. This study follows TRIPOD guidelines (18).

## Results

### Clinicopathologic characteristics

**Figure 1:**
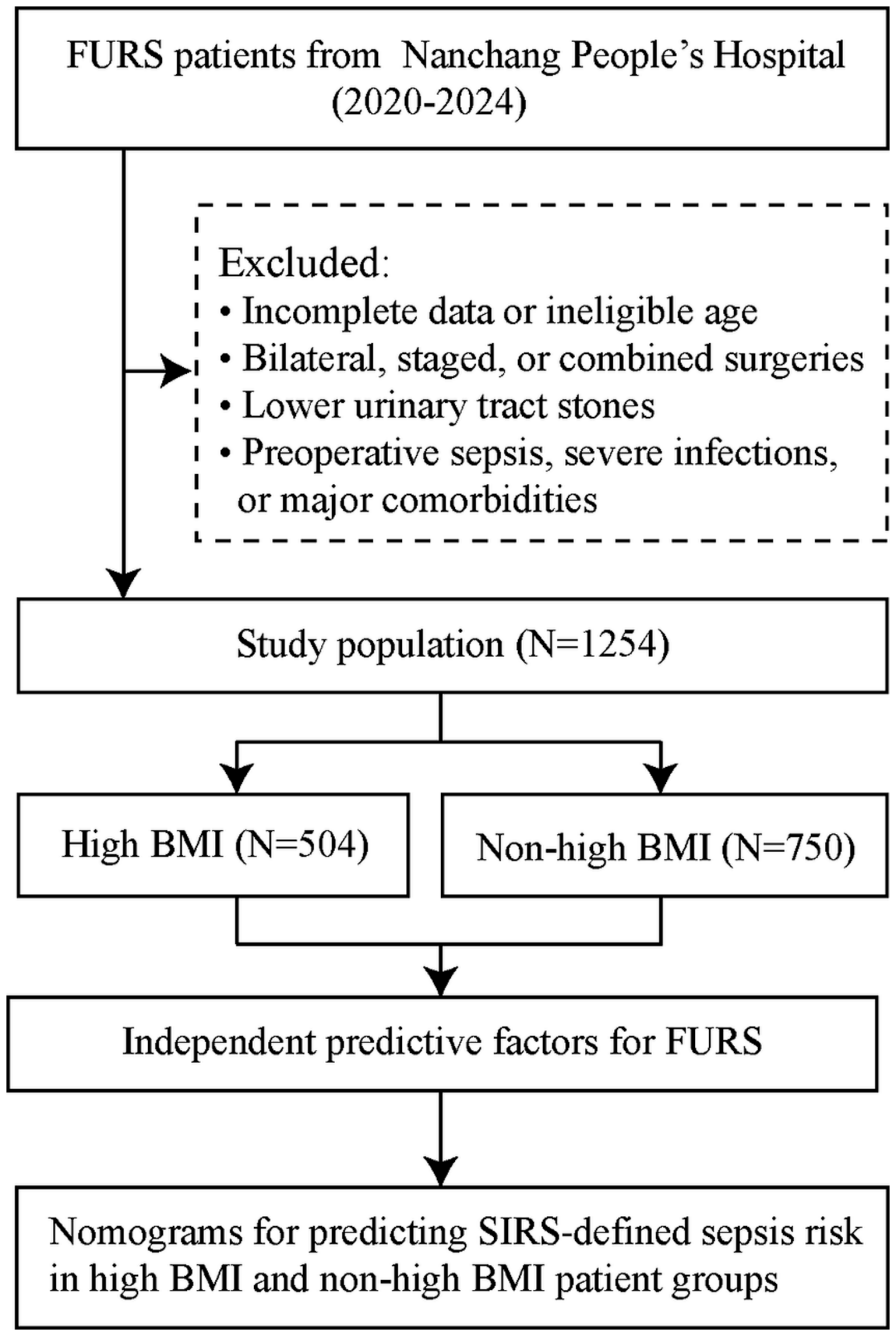
Flowchart of patient selection and BMI stratification.

Based on the inclusion and exclusion criteria, a total of 1,254 patients who underwent FURS were included in the study (Figure 1). Among them, 504 (40.2%) patients were classified as BMI ≥ 25 kg/m^2^ and 750 (59.8%) as BMI < 25 kg/m^2^. Postoperative SIRS-defined sepsis occurred in 78 patients (6.2%), including 42 (8.3%) in the BMI ≥ 25 kg/m^2^ group and 36 (4.8%) in the BMI < 25 kg/m^2^ group (*p* = 0.011).

Baseline characteristics stratified by BMI groups are summarized in Table 1. Compared with patients with BMI < 25 kg/m^2^, those with BMI ≥ 25 kg/m^2^ had higher prevalences of diabetes (*p* = 0.001), hypertension (*p* = 0.004), hyperlipidemia (*p* = 0.005), history of urinary tract infection (*p* = 0.003), pre-stented use (*p* = 0.002), positive preoperative urine culture (*p* < 0.001), larger stone size (*p* = 0.019), staghorn stones (*p* = 0.006), operative time ≥60 min (p < 0.001), elevated CRP ≥ 10 mg/L (*p* = 0.001), and lower postoperative albumin (p < 0.001). No significant difference in preoperative PCT ≥ 0.5 ng/mL was observed between BMI groups (*p* = 0.185).

**Table 1:** Baseline characteristics of patients undergoing FURS, stratified by BMI (≥25 vs. < 25 kg/m^2^).

Variables	Total *N* = 1,254 (%)	BMI ≥25 kg/m^2^ *N* = 504 (%)	BMI <25 kg/m^2^ *N* = 750 (%)	*p*
Gender				0.445
Male	713 (56.9)	280 (55.6)	433 (57.7)	
Female	541 (43.1)	224 (44.4)	317 (42.3)	
Age (yrs)				0.190
< 60	828 (66.0)	322 (63.9)	506 (67.5)	
≥ 60	426 (34.0)	182 (36.1)	244 (32.5)	
Smoking				0.305
Yes	373 (29.7)	158 (31.3)	215 (28.7)	
No	881 (70.3)	346 (68.7)	535 (71.3)	
Diabetes				0.001
Yes	137 (10.9)	73 (14.5)	64 (8.5)	
No	1,117 (89.1)	431 (85.5)	686 (91.5)	
Hypertension				0.004
Yes	277 (22.1)	132 (26.2)	145 (19.3)	
No	977 (77.9)	372 (73.8)	605 (80.7)	
Hyperlipidemia				0.005
Yes	229 (18.3)	111 (22.0)	118 (15.7)	
No	1,025 (81.7)	393 (78.0)	632 (84.3)	
History of UTI				0.003
Yes	220 (17.5)	108 (21.4)	112 (14.9)	
No	1,034 (82.5)	396 (78.6)	638 (85.1)	
Pre-stented				0.002
Yes	83 (6.6)	47 (9.3)	36 (4.8)	
No	1,171 (93.4)	457 (90.7)	714 (95.2)	
Urine culture				<0.001
Positive	114 (9.1)	67 (13.3)	47 (6.3)	
Negative	1,140 (90.9)	437 (86.7)	703 (93.7)	
Stone size (cm)				0.019
< 2	930 (74.1)	356 (70.6)	574 (76.5)	
≥ 2	324 (25.9)	148 (29.4)	176 (23.5)	
Staghorn stone				0.006
Yes	251 (20.0)	120 (23.8)	131 (17.5)	
No	1,003 (80.0)	384 (76.2)	619 (82.5)	
Location of stone				0.435
Left	489 (39.0)	206 (40.9)	283 (37.7)	
Right	450 (35.9)	171 (33.9)	279 (37.2)	
Bilateral	315 (25.1)	127 (25.2)	188 (25.1)	
Number of stones				0.229
Solitary	389 (31.0)	166 (32.9)	223 (29.7)	
Multiple	865 (69.0)	338 (67.1)	527 (70.3)	
Operative time (min)				<0.001
< 60	890 (71.0)	328 (65.1)	562 (74.9)	
≥ 60	364 (29.0)	176 (34.9)	188 (25.1)	
CRP (mg/L)				0.001
< 10	1,100 (87.7)	424 (84.1)	676 (90.1)	
≥ 10	154 (12.3)	80 (15.9)	74 (9.9)	
PCT ng/mL				0.185
< 0.5	1,148 (91.5)	455 (90.3)	693 (92.4)	
≥ 0.5	106 (8.5)	49 (9.7)	57 (7.6)	
Postoperative albumin				<0.001
< 35 g/L	230 (18.3)	59 (11.7)	171 (22.8)	
≥ 35 g/L	1,024 (81.7)	445 (88.3)	579 (77.2)	
SIRS-defined sepsis				0.011
Yes	78 (6.2)	42 (8.3)	36 (4.8)	
No	1,176 (93.8)	462 (91.7)	714 (95.2)	

BMI, body mass index; UTI, urinary tract infection; CRP, C-reactive protein; PCT, procalcitonin; SIRS, systemic inflammatory response syndrome.

Within each BMI stratum, univariable analyses showed that SIRS-defined sepsis was associated with a broadly similar risk profile. In the BMI ≥ 25 kg/m^2^ group, patients who developed SIRS-defined sepsis were more likely to be female, have diabetes, positive urine culture, stone size ≥2 cm, staghorn stones, operative time ≥60 min, elevated CRP, and elevated PCT (all *p* < 0.05). Among BMI < 25 kg/m^2^ patients, SIRS-defined sepsis was significantly associated with positive urine culture, larger stone size, staghorn stones, postoperative albumin <35 g/L, elevated CRP, and elevated PCT (all p < 0.05).

### Independent predictive factors for sepsis

Multivariable logistic regression analysis revealed distinct predictors of SIRS-defined sepsis for patients with BMI ≥ 25 kg/m^2^ and BMI < 25 kg/m^2^ (Table 2). In the BMI ≥ 25 kg/m^2^ group, the following variables were independently associated with SIRS-defined sepsis: positive urine culture (OR = 8.85, 95% CI: 5.94–9.86, *p* < 0.001), presence of staghorn stone (OR = 2.98, 95% CI: 1.07–8.25, *p* = 0.036), operative time ≥60 min (OR = 3.18, 95% CI: 1.17–8.64, *p* = 0.023), elevated CRP ≥ 10 mg/L (OR = 4.39, 95% CI: 1.34–8.80, *p* < 0.001), and elevated PCT ≥ 0.5 ng/mL (OR = 4.65, 95% CI: 1.39–9.79, *p* < 0.001).

**Table 2:** BMI-stratified independent predictors of SIRS-defined sepsis and corresponding nomogram points.

BMI ≥25 kg/m^2^				BMI <25 kg/m^2^			
Predictor	OR (95% CI)	*p*-value	Points	Predictor	OR (95% CI)	*p*-value	Points
Positive urine culture	8.85 (5.94–9.86)	<0.001	100	Positive urine culture	9.93 (5.98–12.45)	<0.001	100
Staghorn stone	2.98 (1.07–8.25)	0.036	37	Staghorn stone	3.98 (1.52–10.44)	0.005	43.5
Operative time ≥60 min	3.18 (1.17–8.64)	0.023	39	—	—	—	—
CRP ≥ 10 mg/L	4.39 (1.34–8.80)	<0.001	90	CRP ≥ 10 mg/L	5.34 (1.45–9.57)	<0.001	75.5
PCT ≥ 0.5 ng/mL	4.65 (1.39–9.79)	<0.001	92	PCT ≥ 0.5 ng/mL	5.95 (1.51–10.51)	<0.001	92.5
—	—	—	—	Postoperative albumin <35 g/L*	4.35^†^ (1.18–16.00)	0.033	63

—Variable not retained in the final multivariable model for that BMI stratum. *Postoperative albumin <35 g/L is presented as the risk factor (inverse of the protective effect of albumin ≥35 g/L, original OR 0.23). ^†^Approximate OR after inversion; calculated as 1/0.23 = 4.35. To obtain predicted probability, sum points for all present predictors and locate total on the nomogram’s total-points axis. OR, odds ratio; CI, confidence interval; CRP, C-reactive protein; PCT, procalcitonin.

In the BMI < 25 kg/m^2^ group, independent predictors included positive urine culture (OR = 9.93, 95% CI: 5.98–12.45, *p* < 0.001), presence of staghorn stone (OR = 3.98, 95% CI: 1.52–10.44, *p* = 0.005), postoperative albumin ≥35 g/L as a protective factor (OR = 0.23, 95% CI: 0.02–0.85, *p* = 0.033), elevated CRP ≥ 10 mg/L (OR = 5.34, 95% CI: 4.04–9.57, p < 0.001), and elevated PCT ≥ 0.5 ng/mL (OR = 5.95, 95% CI: 3.81–10.51, p < 0.001).

Notably, operative time ≥60 min was independently associated with SIRS-defined sepsis only in the BMI ≥ 25 kg/m^2^ group, whereas in the BMI < 25 kg/m^2^ group the association did not reach statistical significance. This stratified difference suggests a potential interaction effect between BMI and operative time on the risk of SIRS-defined sepsis, highlighting the need for subgroup-specific modeling. To formally test whether BMI modifies the effect of the included predictors, we performed an interaction analysis by including BMI × operative time interaction terms in the pooled multivariable logistic regression model. The interaction term between BMI (≥25 vs. < 25 kg/m^2^) and operative time (≥60 vs. < 60 min) was statistically significant (P for interaction = 0.031), supporting the BMI-stratified modeling approach.

### Development and internal validation of the nomogram

**Figure 2:**
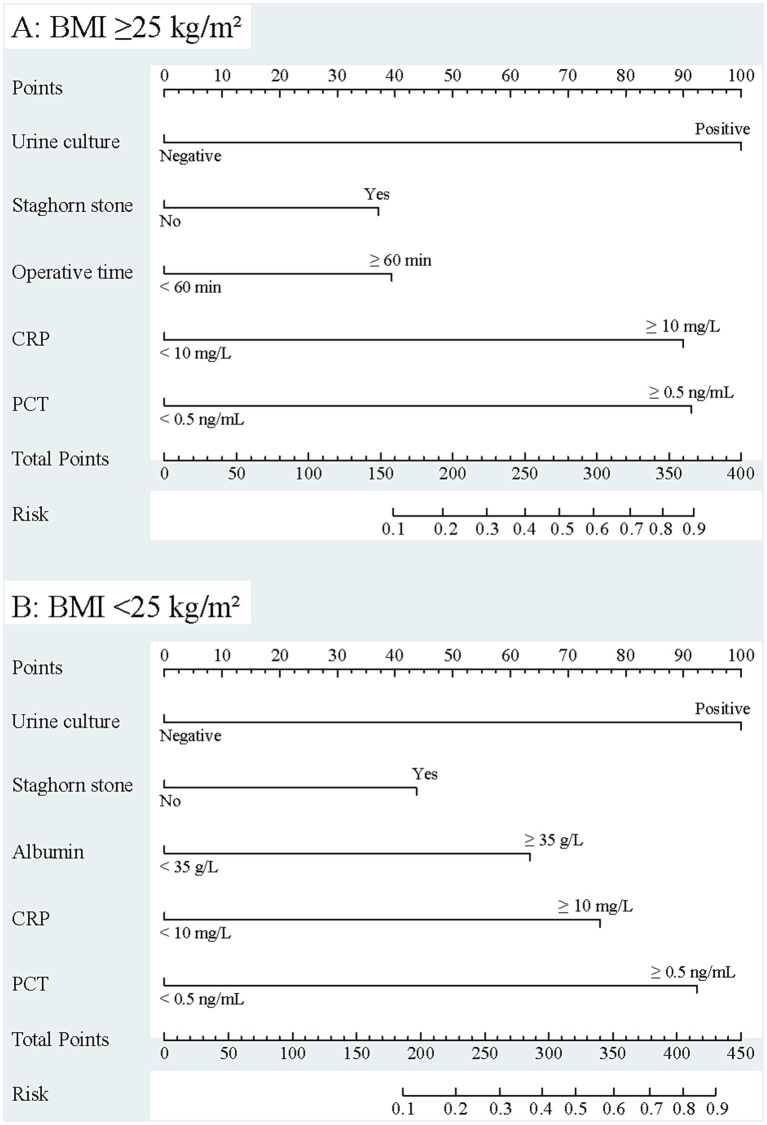
BMI-stratified nomograms for predicting early postoperative SIRS-defined sepsis after FURS in patients with **(A)** BMI ≥ 25 kg/m^2^ and **(B)** BMI < 25 kg/m^2^.

The BMI ≥ 25 kg/m^2^ nomogram incorporated preoperative urine culture, staghorn stones, preoperative CRP ≥ 10 mg/L, preoperative PCT ≥ 0.5 ng/mL, and intraoperative operative time ≥60 min (Figure 2A), whereas the BMI < 25 kg/m^2^ nomogram included preoperative urine culture, staghorn stones, preoperative CRP, preoperative PCT, and early postoperative albumin (within 24 h) (Figure 2B).

A corresponding scoring system was developed based on these nomograms for both BMI strata, with point assignments shown in Table 2 (rightmost columns). In the BMI ≥ 25 kg/m^2^ group, points were: positive urine culture (100), staghorn stone (37), operative time ≥60 min (39), CRP ≥ 10 mg/L (90), and PCT ≥ 0.5 ng/mL (92). For BMI < 25 kg/m^2^ patients, points were: positive urine culture (100), staghorn stone (43.5), CRP ≥ 10 mg/L (75.5), PCT ≥ 0.5 ng/mL (92.5), and postoperative albumin <35 g/L (63). Operative time was not retained in the BMI < 25 kg/m^2^ model. To obtain an individual’s risk, points for each predictor are summed and the total is mapped to the nomogram’s total-points axis to read the predicted probability.

**Figure 3:**
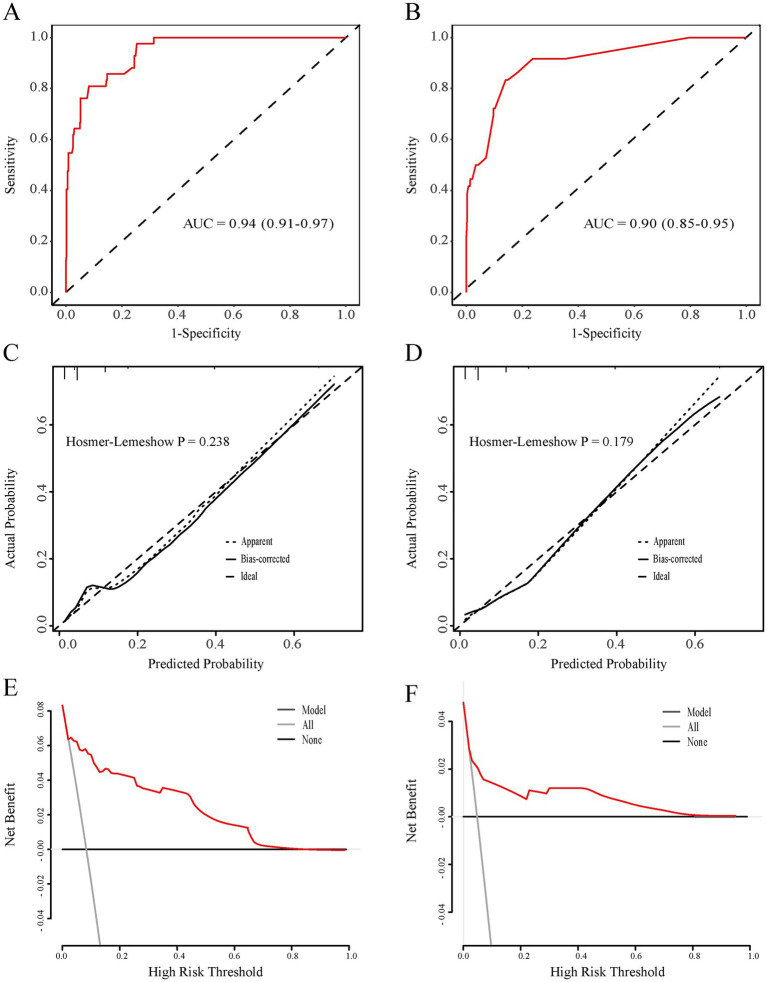
Model validation using ROC curves **(A,B)**, calibration plots **(C,D)**, and decision curve analysis (DCA) **(E,F)** for BMI ≥ 25 kg/m^2^ and BMI < 25 kg/m^2^ groups.

On internal validation, the BMI ≥ 25 kg/m^2^ nomogram demonstrated excellent discrimination, with an apparent AUC of 0.94 (95% CI: 0.91–0.97) and an optimism-corrected AUC of 0.92 (95% CI: 0.89–0.95) (Figure 3A). The BMI < 25 kg/m^2^ nomogram showed similarly robust discrimination, with an apparent AUC of 0.90 (95% CI: 0.85–0.95) and an optimism-corrected AUC of 0.88 (95% CI, 0.83–0.93) (Figure 3B). Bootstrap-corrected calibration slopes were 0.90 for the BMI ≥ 25 model and 0.88 for the BMI < 25 model, with Brier scores of 0.054 and 0.035, respectively, indicating good calibration and providing further reassurance against major overfitting.

Calibration curves showed good agreement between predicted and observed probabilities of SIRS-defined sepsis for both nomograms (Figures 3C,D). Hosmer–Lemeshow tests indicated no evidence of poor fit (*p* = 0.238 for the BMI ≥ 25 kg/m^2^ model and *p* = 0.179 for the BMI < 25 kg/m^2^ model). Additional internal validation metrics are presented in Supplementary Table S1.

DCA (Figures 3E,F) demonstrated that both nomograms yielded positive net benefit across a wide range of clinically relevant threshold probabilities (approximately 10–70%), compared with treat-all or treat-none strategies. These findings suggest that applying the BMI-stratified nomograms in clinical practice may reduce unnecessary interventions while effectively identifying patients at high risk of SIRS-defined sepsis.

## Discussion

In this single-center retrospective cohort, we developed and internally validated BMI-stratified nomograms to predict early SIRS-defined sepsis after FURS. Across both BMI strata, positive preoperative urine culture, staghorn calculi, elevated CRP, and elevated PCT were independent predictors. Operative time ≥60 min was a risk factor only in the BMI ≥ 25 kg/m^2^ group, while postoperative albumin ≥35 g/L was protective only in the BMI < 25 kg/m^2^ group. These findings support a BMI-stratified approach rather than treating BMI as a single covariate. Formal interaction testing further supported this approach, as the BMI × operative time interaction term was statistically significant (P for interaction = 0.031), confirming that BMI significantly modifies the effect of operative time on the risk of SIRS-defined sepsis.

Positive preoperative urine culture showed a strong and similar association with postoperative SIRS defined sepsis in both BMI strata (OR 8.85 in BMI ≥ 25 kg/m^2^; OR 9.93 in BMI < 25 kg/m^2^), highlighting the central role of infection control before FURS. This is consistent with prior reports linking preoperative bacteriuria to infectious complications after endourological procedures (9, 19, 20). Our data reinforce the importance of universal preoperative urine culture screening, timely culture-directed antibiotic treatment, and deferral of elective FURS until clinical control and, whenever feasible, culture clearance, regardless of BMI.

Beyond screening and treatment, two additional considerations merit attention. First, the emergence of antimicrobial resistance among uropathogens in stone patients has become a growing concern; studies have shown that multidrug-resistant organisms, particularly ESBL-producing *Escherichia coli*, are increasingly prevalent in this population (21). Such resistance patterns may compromise the efficacy of standard prophylactic regimens. Second, antimicrobial stewardship principles—emphasizing culture-directed therapy, avoidance of unnecessary prolonged antibiotic exposure, and adherence to institutional antibiogram-guided prophylaxis—should be integrated into perioperative care (22). While our nomograms provide individualized risk stratification, they are intended to complement, rather than replace, sound antimicrobial stewardship practices.

Staghorn calculi were independent risk factors in both strata (BMI ≥ 25 kg/m^2^: OR 2.98; BMI < 25 kg/m^2^: OR 3.98), likely reflecting greater bacterial burden within complex stone architecture and increased procedural difficulty (23). These results support meticulous preoperative assessment of stone burden and individualized operative planning, including consideration of staged procedures in patients with large or staghorn stones (24, 25). Although many stones in this cohort were <2 cm, the presence of partial staghorn calculi, a notable proportion of positive preoperative cultures, and the use of a SIRS-defined endpoint probably contributed to the observed SIRS-defined sepsis incidence. In daily practice, strategies such as culture-directed deferral, drainage-first approaches for infected obstruction, limiting operative time, biomarker screening (CRP/PCT), and selective staging of prolonged cases may collectively mitigate SIRS defined sepsis risk (1). Notably, operative time ≥60 min remained independently associated with SIRS-defined sepsis in patients with BMI ≥ 25 kg/m^2^ despite these measures.

Operative time ≥60 min was an independent predictor only in the BMI ≥ 25 kg/m^2^ group. While this may coincide with greater technical difficulty in some high-BMI cases, operative duration primarily reflects overall stone complexity (2). Prolonged operative time is an established infection risk and warrants attention in all patients (5, 26, 27). In our cohort, the association was statistically significant in the BMI ≥ 25 kg/m^2^ group and directionally similar but non-significant in the BMI < 25 kg/m^2^ group, likely due to fewer events and residual confounding. We therefore interpret operative time as a modifiable intraoperative marker—prompting time-based staging and closer postoperative observation—rather than a BMI-specific causal effect. Procedural duration should be minimized irrespective of BMI.

Preoperative CRP was associated with higher postoperative SIRS defined sepsis risk in both BMI strata. As a widely used marker of systemic inflammation, CRP has been shown to predict infectious complications across multiple surgical settings, including hepatobiliary and orthopedic surgery (28, 29). Our findings extend these observations to FURS and support incorporating CRP into routine preoperative risk stratification, especially when interpreted alongside urine culture and clinical signs of infection. Similarly, PCT—an infection-specific biomarker—predicted postoperative SIRS-defined sepsis in both strata (BMI ≥ 25 kg/m^2^: OR 4.65; BMI < 25 kg/m^2^: OR 5.95). Because it rises rapidly in bacterial infection and can outperform CRP in distinguishing bacterial from non-bacterial inflammation (30), preoperative PCT has clinical relevance in endourology; elevated levels have been linked to post-procedure sepsis after endourological interventions (31). These data support incorporating PCT into perioperative risk assessment to guide early antibiotic decisions and tighten infection-control strategies.

It is important to clarify the intended timing of model application. The BMI ≥ 25 kg/m^2^ nomogram, which includes operative time as a predictor, is designed for application at case completion—once operative duration is known—to guide postoperative monitoring intensity and escalation of care. The BMI < 25 kg/m^2^ nomogram, which includes postoperative albumin measured within 24 h after surgery, is likewise intended for early postoperative risk stratification. We acknowledge that postoperative albumin may be influenced by the early inflammatory response; therefore, this model is not intended for preoperative decision-making but rather for refining risk estimates in the early postoperative period to inform clinical management.

Postoperative albumin was an independent protective factor in the BMI < 25 kg/m^2^ group. Hypoalbuminemia reflects poor nutritional and inflammatory status and is linked to higher postoperative infection and worse outcomes (32). In patients with BMI < 25 kg/m^2^, low albumin may indicate limited physiological reserve and reduced immune competence. Prior studies in urological and abdominal surgery show that maintaining serum albumin improves prognosis (33, 34). These findings support routine perioperative albumin monitoring and early nutritional optimization in this subgroup.

The BMI-stratified nomograms performed well (AUC 0.94 for BMI ≥ 25; 0.90 for BMI < 25). Decision-curve analysis showed net benefit for predicted risks of 10–70%. A risk ≥20% may prompt escalated prevention (close observation, early labs, antibiotic reassessment), while <10% supports routine care. As noted above, the intended timing of application differs between the two models: the BMI ≥ 25 nomogram, which incorporates intraoperative operative time, can be applied immediately at case completion; the BMI < 25 nomogram, which includes postoperative albumin, is designed for use within 24 h after surgery to refine risk estimates and guide early postoperative management. CRP and PCT are available preoperatively, operative time intraoperatively, and albumin (only in the BMI < 25 model) postoperatively. The nomograms can be applied at case completion to guide monitoring and staging; for lower-BMI patients, risk estimates can be refined within 24 h postoperatively.

Our findings support prior work linking higher BMI to postoperative infection risk (27, 35). The key advance is BMI-stratified modeling, which revealed operative time ≥60 min as a risk factor only in the BMI ≥ 25 kg/m^2^ group, encouraging shorter procedures and time-based staging in these patients. BMI may also increase susceptibility through chronic inflammation, immune dysregulation, and altered antibiotic pharmacokinetics—mechanisms warranting prospective study.

This study has several limitations. First, the endpoint was SIRS defined (not Sepsis 3) due to incomplete SOFA/qSOFA data, allowing possible misclassification. Second, event numbers were modest after BMI stratification, with events per variable of 8.4 and 7.2 for the two nomograms, respectively; although bootstrap validation suggested robust performance and the optimism-corrected calibration slopes were close to 1.0 (0.94 and 0.91), we acknowledge that the limited events may affect model stability, and the high AUC values should be interpreted with caution. External validation is essential before clinical implementation. Third, detailed univariable analysis results and missing data summaries were not presented due to space constraints, which we acknowledge as a limitation. Fourth, dichotomization using conventional thresholds (e.g., WHO BMI 25 kg/m^2^) may not capture Asian specific or non linear effects. Fifth, unmeasured variables (stent dwell time, intrarenal pressure, sheath type, antibiotic regimens) may cause residual confounding. Finally, single center design limits generalizability; multicenter external validation and local recalibration are critically needed before clinical implementation, and we plan to address this in future studies.

## Conclusion

In this study, we developed and internally validated BMI-stratified nomograms for predicting SIRS-defined sepsis after FURS, identifying both shared and BMI-specific risk factors. These findings support a stratified approach to perioperative risk assessment, particularly for patients with BMI ≥ 25 kg/m^2^. While the nomograms may facilitate individualized risk stratification, external multicenter validation and prospective impact studies are necessary before routine clinical application.

## Data Availability

The raw data supporting the conclusions of this article will be made available by the authors, without undue reservation.
