# Supplementary material for: BMI-stratified nomograms to predict early SIRS-defined sepsis after flexible ureteroscopy
Source: Front Med (Lausanne). 2026 Jul 16;13:1855137. doi: 10.3389/fmed.2026.1855137 (PMC13422523; doi:10.3389/fmed.2026.1855137)
Supplement: Supplementary file 1 [file Table_1.DOCX]

**Supplementary Table S1.** Internal validation metrics for BMI-stratified prediction models.

| **Model** | **Apparent AUC** | **Optimism-corrected AUC** | **Bootstrap-corrected calibration slope** | **HL P-value** | **Brier score** |
| --- | --- | --- | --- | --- | --- |
| BMI ≥25 kg/m² | 0.94 | 0.92 | 0.90 | 0.238 | 0.054 |
| BMI <25 kg/m² | 0.90 | 0.88 | 0.88 | 0.179 | 0.035 |

AUC, area under the curve. Optimism-corrected estimates were obtained from 1,000 bootstrap resamples. Calibration slopes and Brier scores are bootstrap-corrected. Hosmer–Lemeshow test P > 0.05 indicates adequate calibration.
